# MicroRNA-145 Protects against Myocardial Ischemia Reperfusion Injury via CaMKII-Mediated Antiapoptotic and Anti-Inflammatory Pathways

**DOI:** 10.1155/2019/8948657

**Published:** 2019-09-10

**Authors:** Zhebo Liu, Bo Tao, Suzhen Fan, Yong Pu, Hao Xia, Lin Xu

**Affiliations:** ^1^Department of Cardiology, Renmin Hospital of Wuhan University, Wuhan 430060, China; ^2^Cardiovascular Research Institute, Wuhan University, Wuhan 430060, China; ^3^Hubei Key Laboratory of Cardiology, Wuhan, China; ^4^Renmin Hospital of Hannan District, Renmin Hospital of Wuhan University, Wuhan, China

## Abstract

MicroRNA-145 (miR-145) has been shown to play an important role in cardiovascular system disorders; however, the underlying mechanism is not completely understood. The purpose of this study was aimed at elucidating the cardioprotective effects of miR-145 against myocardial ischemia/reperfusion (I/R) injury. We established a rat myocardial I/R model with 45 min left anterior descending coronary artery (LAD) occlusion and 2 h reperfusion. The levels of myocardial enzymes, apoptotic, inflammatory, and oxidative indices were determined. The arrhythmia score was assessed by programmed electrical stimulation (PES). Quantitative real-time PCR and western blot were applied to evaluate the expression levels of miR-145 and related target proteins, respectively. I/R injury decreased the expression of miR-145; however, upregulated miR-145 markedly reduced the elevation of ST segment, decreased corrected QT (QTc) intervals, and attenuated I/R-induced electrophysiological instability. Furthermore, miR-145 suppressed myocardium apoptotic, inflammatory, and oxidative response as well as the phosphorylation of Ca^2+^/calmodulin-dependent protein kinase II (CaMKII), ryanodine receptor2 (RyR2 Ser2814), apoptosis signal-regulating kinase 1 (ASK1), c-Jun NH2-terminal kinases (JNK), and nuclear translocation of nuclear factor kappa-B (NF-*κ*B) p65. In summary, overexpression of miR-145 alleviates I/R-induced myocardial electrophysiological instability and apoptotic and inflammatory response via inhibition of the CaMKII-mediated ASK1 antiapoptotic pathway and NF-*κ*B p65 anti-inflammatory pathways.

## 1. Introduction

Acute myocardial infarction (AMI) is one of the leading causes of deaths worldwide and is predicted to be a major threat to human health [1, 2]. Ischemia/reperfusion (I/R) injury is an acute adverse cardiac event characterized by cardiomyocyte apoptosis, inflammatory response, and oxidative stress following AMI [3]. In addition, IR injury further aggravates myocardial malfunction, induces arrhythmia, and worsens the prognosis of patients [4]. Therefore, it is crucial to explore an effective strategy for the prevention of IR injury.

MicroRNAs (miRs) refer to a class of noncoding RNA 18 to 25 nucleotide long. miRs are usually assembled into RNA-induced silencing complexes and, thus, negatively regulate the translation of mRNA [5]. They influence a wide range of biological functions, and studies have shown that miRs exert a vital role in cardiac remodeling [6]. For instance, miR-9 negatively regulates cardiac hypertrophy by targeting myocardin which has been demonstrated to be a promoter of cardiac hypertrophic responses [7]. Restoration of miR-24 expression to physiological levels in AMI models has been proven to effectively attenuate cardiomyocyte apoptosis and decrease scar size [8].

miR-145 is abundantly expressed in the cardiovascular system. Previous studies on miR-145 mainly focused on diseases such as atherosclerosis and tumor; however, its role in cardiovascular diseases has been rarely reported. Recently, Higashi et al. [9] found that the expression of miR-145 decreased in rats with myocardial infarction, whereas upregulation of miR-145 expression facilitated the repair of an infarcted myocardium. Furthermore, Yuan et al. [10] proved that miR-145 exerted a cardioprotective effect in myocardial I/R injury by ameliorating inflammation via regulation of CD40 in vitro. Moreover, prior studies [9, 11] have demonstrated that antagonizing miR-145 pretreatment significantly aggravated myocardial I/R injury by deteriorating infarcted myocardium size, apoptosis, and inflammation in mouse. Although miR-145 may represent a novel potential therapeutic target, its role in cardiac I/R injury in vivo is still unclear.

CaMKII is a serine/threonine protein kinase with multiple functions and was proven to be a potential target of miR-145 [12]. Numerous evidences have shown that appropriate phosphorylation of CaMKII was necessary for the mechanical recovery of an I/R-injured myocardium. However, under a prolonged ischemic period, the overactivated CaMKII could further aggravate I/R injury by increasing apoptosis [13]. ASK1 and NF-*κ*B p65 have been demonstrated to exert significant detrimental effects in I/R pathophysiological process, manifested by promoting apoptosis and inflammation response, which were abrogated by ASK1/NF-*κ*B p65 inhibition [14, 15]. A previous study by Kashiwase et al. [16] showed that ASK1 and NF-*κ*B p65 appeared to act downstream of CaMKII. For these reasons, we hypothesized that miR-145 could alleviate I/R injury via CaMKII-mediated ASK1 and NF-*κ*B p65 signaling cascades.

In this study, we established I/R models with an extended ischemia period (45 min) and apoptotic, oxidative stress, and inflammatory response were evaluated to investigate the effects of miR-145 on myocardial I/R injury. In addition, we examined the potential modulatory mechanism of miR-145 on CaMKII signaling cascades.

## 2. Material and Methods

### 2.1. Animals

Male SD rats aged 7-8 weeks (210-230 g) were acclimatized to laboratory conditions, maintained under controlled temperature (24 ± 2°C), and subjected to a natural photoperiod (12 h light/dark cycle). The rats were allowed free access to food and water during the entire experiment period. All procedures involving animals were conducted in accordance with Capital Medical University and the Use of Laboratory Animals guidelines published by the US National Institutes of Health (NIH Publication, revised 1996) and were approved by the Institutional Animal Care and Use Committee of Wuhan University.

### 2.2. Myocardial I/R Model, IPC Model, and Experimental Design

Rats were anesthetized by intraperitoneal injection of pentobarbital (50 mg/kg) and fixed for endotracheal intubation using a small animal respirator. Artificial respiration was provided by a respirator with a frequency of 70 strokes/min and a tidal volume of 1.5 ml/100 g. Then, the thoracotomy was conducted at the left fourth intercostal space, and the heart was exposed after pericardiotomy. Subsequently, the left anterior descending coronary artery (LAD) was ligated approximately 2 mm below the left atrial appendage with a 6-0 silk suture to induce ischemia. Successful ischemia was confirmed by the elevation of ST segment. The myocardial I/R rat model was established with 45 min LAD occlusion and 2 h reperfusion.

The IPC model was established as previously described [17]. Briefly, rats were subjected to 3 × 5 min of I/R episodes followed by an intervening 10 min period before the induction of prolonged ischemia.

Rats were randomly divided in to five groups: A—sham group, the sham-operated control group; B—I/R group; C—I/R+Ad-Scramble group, transfection of Ad-Scramble 1 week prior to induction of I/R; D—I/R+Ad-miR-145 group, transfection of Ad-miR-145 1 week prior to induction of I/R; and E—I/R+IPC group, conduction of IPC prior to the prolonged I/R.

### 2.3. Transfection of Ad-miR into the Heart of Rat In Vivo

Thoracotomy and pericardiotomy were performed between the third and fourth ribs to expose the heart. A 33-gauge needle was used to inject 100 *μ*l Ad-miR-145 (1 × 10^11^ PFU, GeneChem Co. Ltd., Shanghai) or Ad-Scramble (1 × 10^11^ PFU, GeneChem Co. Ltd., Shanghai) into five sites of the left ventricular (LV) wall. After injection, the chest was stitched, and the rat was allowed to recover subsequently.

### 2.4. Quantitative Real-Time PCR

Total RNA was isolated from the heart tissue using a TRIzol Reagent (Life Technologies). Subsequently, a TaqMan MicroRNA reverse transcriptase kit (Applied Biosystems, Bedford, MA) was used for the quantification of miR-145 and U6 control transcripts according to the manufacturer's instructions. The primers used were as follows (5′-3′): miR-145-5p (reverse transcript)—CTCAACTGGTGTCGTGGAGTCGGCAATTCAGTTGAGAGGGATTC, miR-145-5p (forward)—TGTCCAGTTTTCCCAGGAATC, miR-145-5p (reverse)—CTCAACTGGTGTCGTGGAGTC; U6 (reverse transcript)—AACGTTCACGAATTTGCGT, U6 (forward)—CCTGCTTCGGCAGCACAT, U6 (reverse)—AACGCTTCACGAATTTGCGT. The miR-145 expression levels were normalized against U6 levels calculated using the 2^-*ΔΔ*Ct^ method.

### 2.5. Histopathological Examination

The heart tissue was excised and washed with saline solution, fixed in 4% paraformaldehyde, and embedded in paraffin after 2 h reperfusion. Several slices (5 *μ*m thick) of paraffin-embedded hearts were sectioned laterally at the level of LV papillary muscles and stained with hematoxylin and eosin (H&E) stain for morphometric research. Damage scores were classified as follows [18]: 0, with no abnormality; 1, with interstitial edema and focal necrosis; 2, with widespread cardiomyocytes swelling and necrosis; 3, necrosis with contraction bands and leukocytic infiltration; and 4, widespread necrosis with contraction bands, hemorrhage, and leukocytic infiltration.

### 2.6. Measurement of Electrocardiograph Variables

Surface electrodes were placed subcutaneously, and surface-lead electrocardiograph II was continuously recorded during the I/R procedure. The elevation of ST segment and changes in corrected QT (QTc) intervals were measured on a basic state: ischemia for 45 min and reperfusion for 30 min, 60 min, and 120 min. In order to correct the heart rate, the QTc was calculated using Bazett's formula [19]: QTc = QT/(RR/100)^1/2^. The QT interval was measured from the beginning of QRS to the end of T waves. The RR interval was automatically analyzed by LabChart 8.0 (ADInstruments).

### 2.7. Biochemical Analysis of Plasma

Blood samples were collected and centrifuged (3000 rpm × 15 min) after reperfusion for 2 h. Corresponding commercial kits (Jiancheng Bioengineering Institute, China) were used to measure the serum concentration of creatinine kinase-MB (CK-MB) and lactate dehydrogenase (LDH) and the activities of superoxide dismutase (SOD) and malondialdehyde (MDA) according to the manufacturer's instructions.

### 2.8. Detection of Myocardial Apoptosis

Cardiomyocyte apoptosis in the heart tissue was detected by terminal deoxynucleotidyl transferase-mediated dUTP nick end labeling (TUNEL) assay according to the instructions of in situ cell death detection kit (Roche Diagnostics, Germany). Subsequently, a 5 *μ*m thick paraffinized tissue section was costained with hematoxylin, and cardiomyocytes with prominent nuclear labeling were counted as TUNEL-positive cells. Four randomly selected fields (×200 magnification) per heart were analyzed using Image-pro Plus 6.0 (Media Cybernetics, USA).

### 2.9. Programmed Electrical Stimulation (PES)

The heart was exposed as previously described, and PES was completed within 10 min before the end of ischemia and reperfusion at the infarct border zone. The protocol for PES was similar to that described in a previous study [20]. Briefly, a custom-made electrical stimulator was used to induce ventricular arrhythmias, and the pacing involved eight paced beats at a cycle length of 120 ms (S_1_), followed by 1-3 extrastimuli (S_2_, S_3_, and S_4_) at shorter coupling intervals. Ventricular tachyarrhythmias (VT) consisted of sustained VT (VT lasted >15 beats) and nonsustained VT (VT lasted 6-15 beats); the arrhythmia scoring system was classified as shown in Table 1 [21]. The highest score was calculated when multiple arrhythmias were induced.

### 2.10. Immunofluorescence

The distribution of NF-*κ*B p65 in cardiomyocytes was evaluated using immunofluorescence assay. Slices of paraffin-embedded hearts were incubated with 3% H_2_O_2_ for 10 min at room temperature. After incubation with the p65 primary antibody (1 : 200, Proteintech, China) at 4°C overnight, the tissue was washed with PBS thrice for 5 min. The slices were then incubated with a secondary antibody for 50 min at 37°C and DAPI solution for 5 min at room temperature successively. The staining images were obtained using an immunofluorescence microscope (OLYMPUS, Japan) and merged by the MicroPublisher system (QImaging, USA).

### 2.11. Echocardiography

Echocardiography was performed under light anesthesia 2 h after reperfusion using a Vinno 6th ultrasound Doppler imaging system (VINNO6, Vinno Corporation, China). M-mode images from a midpapillary level were obtained to evaluate the following parameters: left ventricular end-diastolic dimension (LVEDd), left ventricular end-systolic dimension (LVEDs), and left ventricular fractional shortening (FS).

### 2.12. Western Blot Analysis

Heart samples were obtained after reperfusion for 2 h and were immediately stored at -80°C before western blot analyses were performed. Protein was extracted using the RIPA protein lysate (ASPEN, Wuhan, China), and the concentration of the protein was determined using the BCA protein concentration assay kit (ASPEN, Wuhan, China). Subsequently, protein (40 *μ*g) was separated by 10% SDS-PAGE and electrotransferred to PVDF membranes. After incubation with corresponding antibodies, the PVDF membranes were then washed by TBST and incubated with a secondary antibody for 1 h at room temperature. A chemiluminescence method was used to detect protein bands, and AlphaEaseFC software processing system (Alpha Innotech, USA) was used to analyze the optical density of target bands. The following primary antibodies were used for western blot analysis: Bax (1 : 2000), p-ASK1 (1 : 500), ASK1 (1 : 1000), NF-*κ*B p65 (1 : 1000), histone H3 (1 : 3000), p-JNK (1 : 1000), and JNK (1 : 2000) (Cell Signaling Technology, USA); Bcl-2 (1 : 2000), caspase 3 (1 : 2000), p-CaMKII (1 : 1000), CaMKII (1 : 500), TNF-*α* (1 : 1000), IL-6 (1 : 500), IL-1*β* (1 : 500), and RyR2 (1 : 500), GAPDH (1 : 10000) (Abcam, UK); ox-CaMKII (1 : 500) (GeneTex, USA); p-RyR2 (Ser2814, 1 : 500) (Badrilla, UK); and cleaved caspase 3 (1 : 1000) (Affbiotech, USA).

### 2.13. Statistical Analysis

Statistical analysis was performed using SPSS 19.0 (IBM, USA). Data was expressed the as the mean ± standard deviation (SD), and statistical differences were evaluated by one-way analysis of variance (ANOVA) or Student's two-tailed *t*-test. A *P* value less than 0.05 was considered to be statistically significant.

## 3. Results

### 3.1. Relative Expression of miR-145

**Figure 1:**
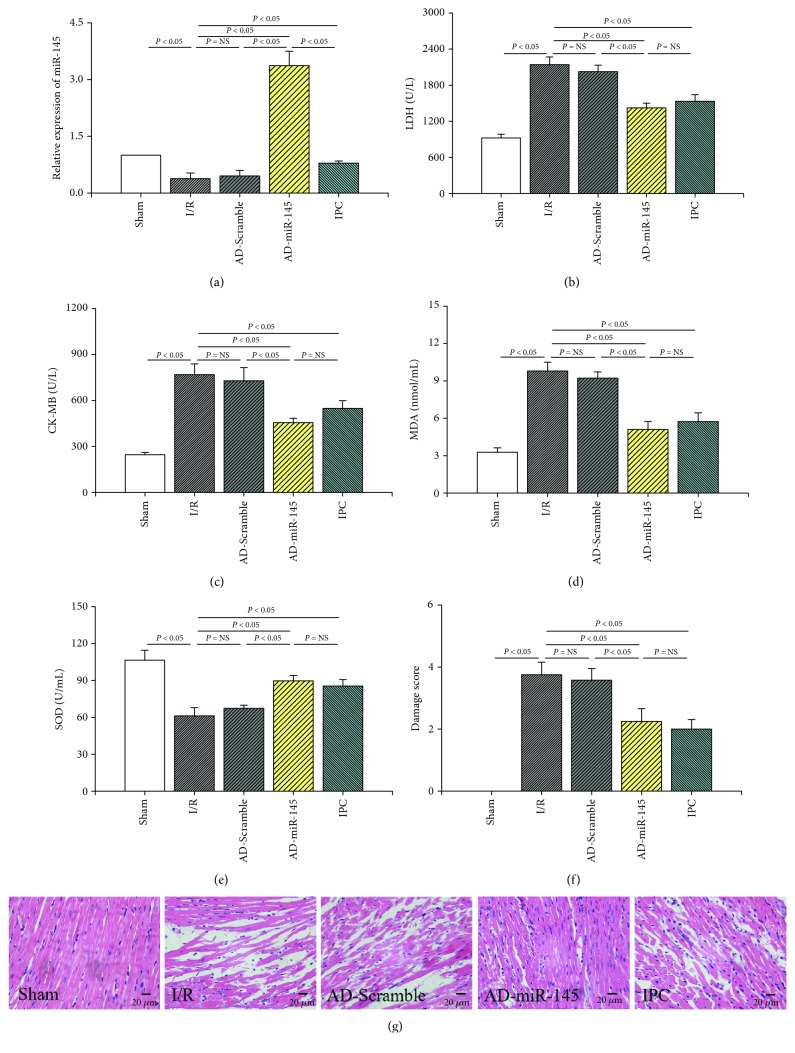
miR-145 attenuated myocardial I/R injury. (a) Relative expression of miR-145 (*n* = 4). (b) The expression of LDH (*n* = 6). (c) The expression of CK-MB (*n* = 6). (d) The contents of MDA (*n* = 6). (e) The activities of SOD (*n* = 6). (f) Damage score (*n* = 6). (g) Representative images of H&E-stained samples (200x magnification).

The expression of miR-145 was detected by quantitative real-time PCR in rats that underwent I/R operation. As shown in Figure 1(a), the level of miR-145 was dramatically downregulated in I/R myocardial tissues, and it was upregulated in AD-miR-145-transfected rats, which also indicated successful transfection of AD-miR-145.

### 3.2. miR-145 Reduced Myocardial Injury after I/R Injury

The leakage of LDH and CK-MB, which indicated injury on the cytomembrane of cardiomyocytes, was significantly increased in the I/R group compared with the sham group (Figures 1(b) and 1(c)). These parameters markedly decreased in the IPC and AD-miR-145-transfected groups. Compared with the sham group, the activity of SOD significantly decreased, and the content of MDA significantly increased in the I/R group (Figures 1(d) and 1(e)). However, SOD activities markedly increased, and MDA contents markedly decreased in the AD-miR-145 group as well as in the IPC group, suggesting that overexpression of miR-145 contributed to the attenuation of myocardial I/R injury.

As shown in Figures 1(f) and 1(g), I/R injury deteriorated a myocardial structure by promoting necrosis, inflammation, and cell infiltration edema. However, the morphological disorder of cardiomyocytes was attenuated in the AD-miR-145 and IPC groups with a notably lower damage score.

### 3.3. Effect of miR-145 on the Elevation of ST Segment and Changes in QTc Interval

**Figure 2:**
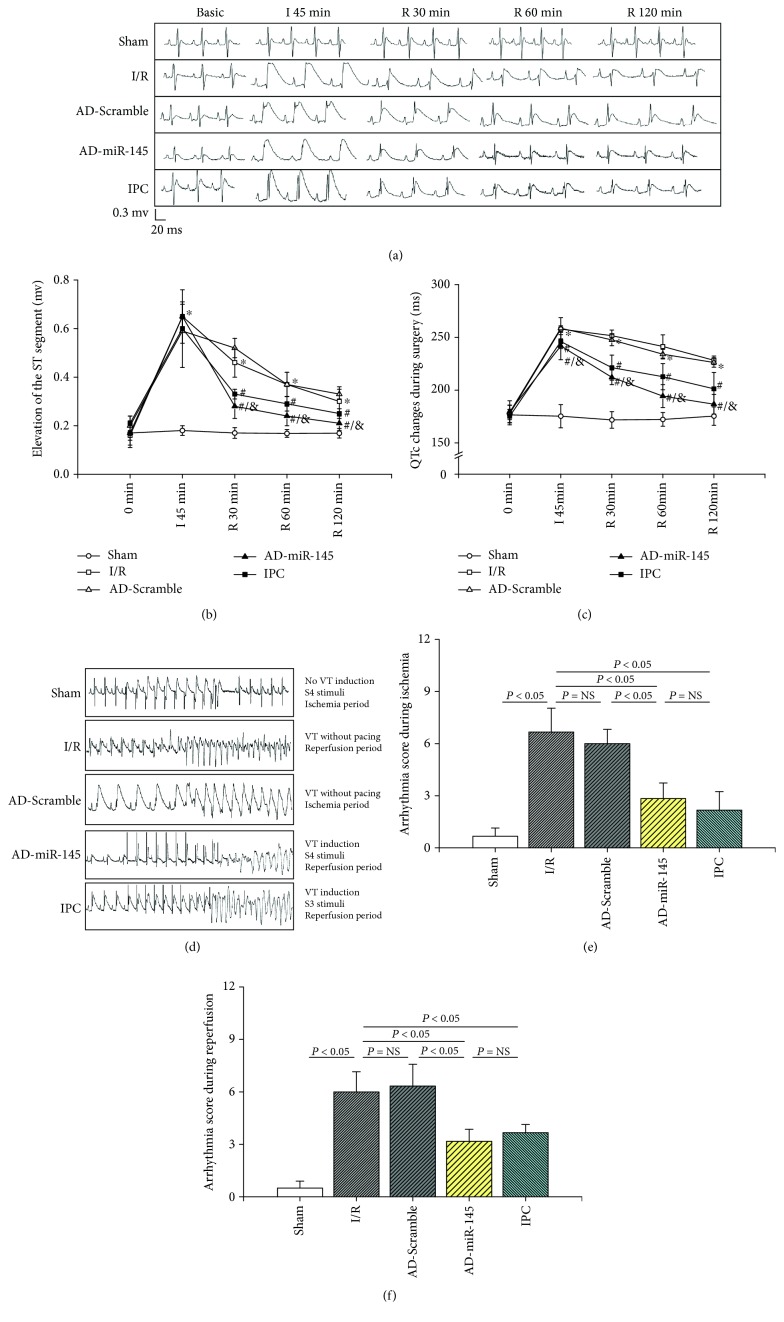
miR-145 suppressed the myocardial I/R injury and susceptibility to VT in an I/R-injured myocardium. (a) Typical segments of ECG on basic; ischemia for 30 min; and reperfusion for 30 min, 60 min, and 120 min. (b) The elevation of ST segment (*n* = 6). (c) The changes of QTc interval (*n* = 6). (d) Examples of PES recordings. (e) Arrhythmia score during ischemia period (*n* = 6). (f) Arrhythmia score during reperfusion period (*n* = 6). I: ischemia; R: reperfusion; VT: ventricular tachyarrhythmias; ^∗^*P* < 0.05 compared with the sham group; ^#^*P* < 0.05 compared with the I/R group; ^&^*P* < 0.05 compared with the AD-Scramble group.

I/R injury remarkably enhanced the elevation of ST segment and prolonged the QTc interval compared with the sham group 45 min after ischemia and 30, 60, and 120 min after reperfusion (Figures 2(a)–2(c)). However, when compared with the I/R group, IPC pretreatment as well as overexpression of miR-145 exhibited a marked inhibitory effect against the elevation of ST and extension of the QTc interval at each observation period during reperfusion, demonstrating that miR-145 overexpression effectively attenuated I/R injury at the onset of reperfusion.

### 3.4. miR-145 Suppressed the Susceptibility of I/R Injured Myocardium to VT

As shown in Figure 2(d), an I/R-injured myocardium was more susceptible to VT, and the arrhythmia score was dramatically higher during ischemia (Figure 2(e)) and reperfusion (Figure 2(f)) periods compared with the sham group. However, I/R-induced susceptibility to VT was ameliorated in the miR-145 and IPC groups. These findings indicated that miR-145 and IPC could simultaneously inhibit the malignant arrhythmia risk of the myocardium during the ischemia and reperfusion periods.

### 3.5. miR-145 Upregulation Suppressed Myocardial Apoptosis

**Figure 3:**
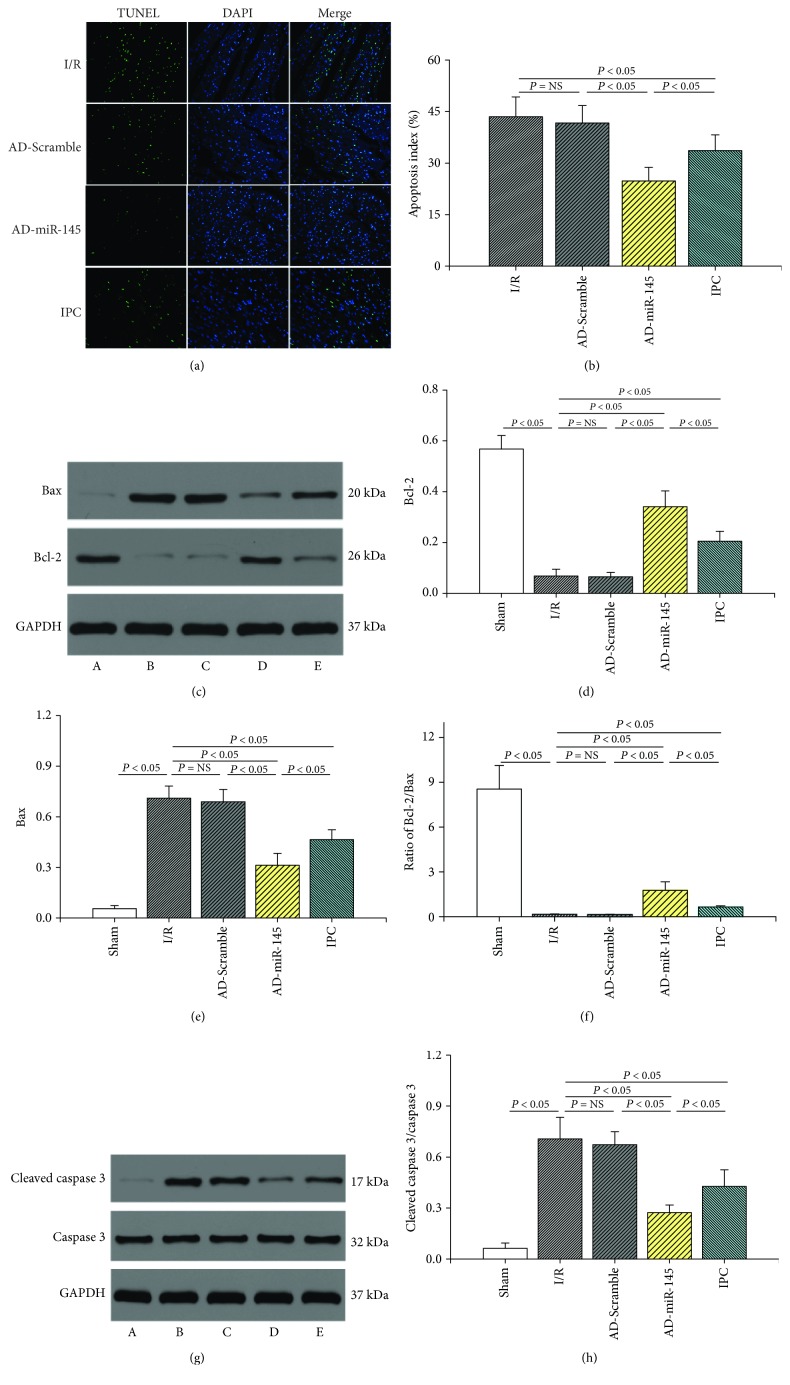
miR-145 upregulation suppressed myocardial apoptosis. (a) Representative images of TUNEL assays (200x magnification). (b) Apoptosis index (*n* = 6). (c) Representative western blots of Bcl-2 and Bax. (d–f) Quantitative analysis of the immunoreactive band displayed by a bar graph (*n* = 3). (g) Representative western blots of cleaved caspase 3 and caspase 3. (H) Quantitative analysis of the immunoreactive band displayed by a bar graph (*n* = 3). (a) Sham group, (b) I/R group, (c) AD-Scramble group, (d) AD-miR-145 group, the (e) IPC group.

TUNEL staining was employed to evaluate the myocardial apoptosis during I/R injury. As shown in Figure 3, the apoptosis index (AI) and the levels of Bax and cleaved caspase 3 dramatically increased in the I/R group. In addition, the expression of Bcl-2 and the ratio of Bcl-2/Bax markedly decreased in the I/R group compared with the sham group. However, when compared with the I/R and IPC groups, upregulated miR-145 substantially decreased AI as well as the levels of Bax and cleaved caspase 3 and significantly increased Bcl-2 expression and the ratio of Bcl-2/Bax. These results suggested that miR-145 suppresses I/R-induced myocardial apoptosis, and such effects were superior to IPC treatment.

### 3.6. Effects of miR-145 on Cardiac Function

**Figure 4:**
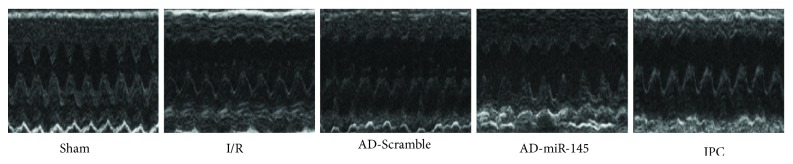
Representative images of echocardiograph.

After 2 h of reperfusion, cardiac performance was analyzed using an echocardiograph. As shown in Figure 4 and Table 2, I/R aggravated cardiac functions, manifested by enlarging LVEDs and decreasing FS; miR-145 and IPC treatment partially attenuated the I/R-induced cardiac dysfunction by increasing FS. These findings were consistent with the results of Higashi et al. [9] who demonstrated that upregulation of miR-145 significantly improved cardiac performance and remodeling after I/R injury.

### 3.7. miR-145 Inhibited CaMKII Signaling Pathway

**Figure 5:**
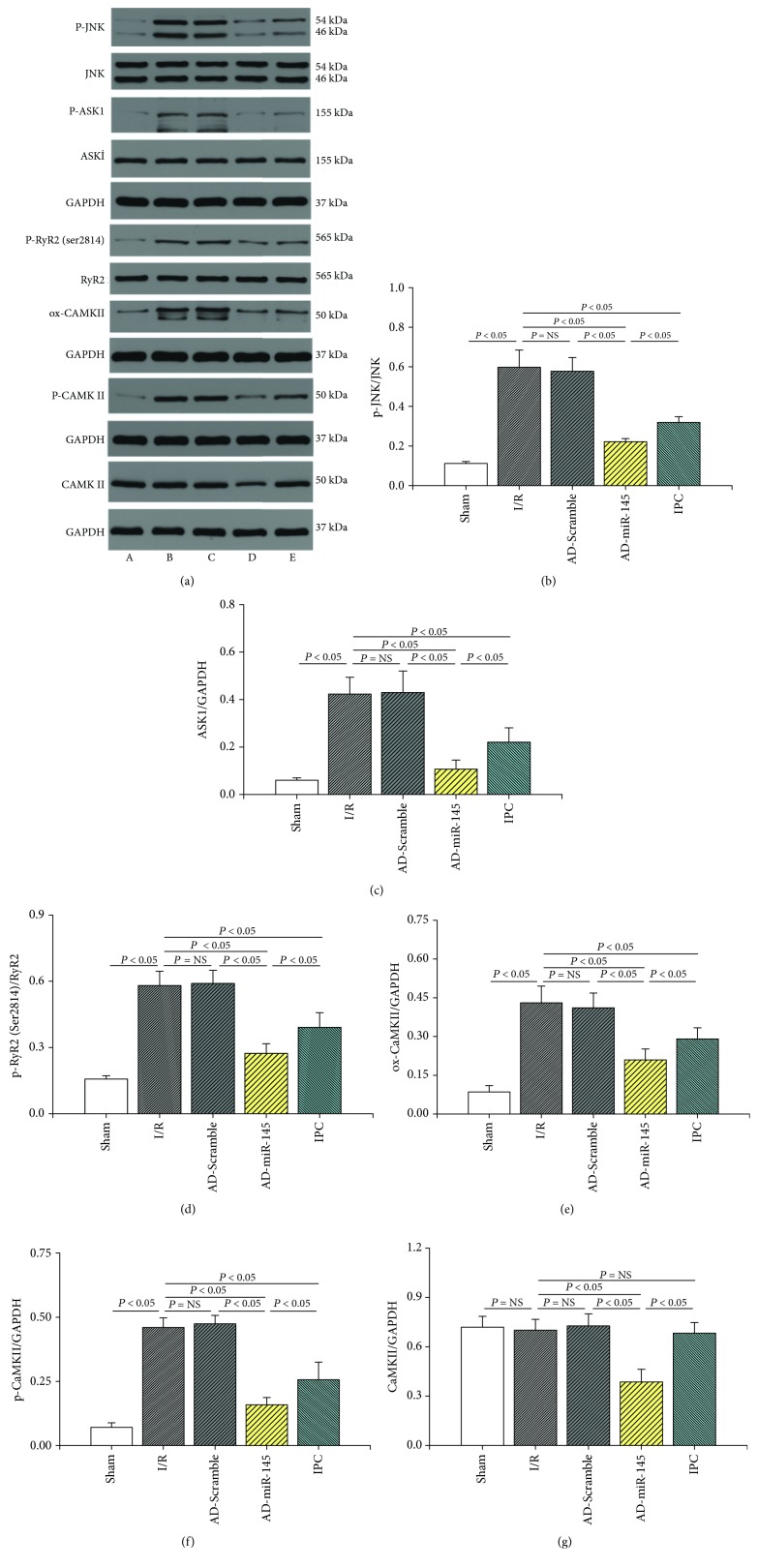
Mechanism underlying cardioprotective effects of miR-145. (a) Representative western blots. (b–g) Quantitative analysis of the immunoreactive band displayed by a bar graph (*n* = 3). (A) Sham group, (B) I/R group, (C) AD-Scramble group, (D) AD-miR-145 group, and (E) IPC group.

In order to elucidate the cardioprotective mechanism of miR-145 in myocardial I/R injury, possible signal pathways were investigated. As shown in Figures 5(a)–5(c) and 5(e)–5(g), substantially, oxidation of CaMKII and phosphorylation of JNK, ASK1, and CaMKII were observed in the I/R group. Meanwhile, upregulated miR-145 expression notably suppressed the expression of total CaMKII and I/R-induced accumulation of p-ASK1, ox-CaMKII, and p-CaMKII, suggesting that miR-145 might exert cardioprotective effects via inhibition of the CaMKII-mediated ASK1/JNK proapoptotic signal pathway. In addition, I/R-induced hyperactivation of RyR2 (Ser2814), a leading cause of myocardium Ca^2+^ oscillations, was also suppressed by miR-145 treatment (Figures 5(a) and 5(d)). These results indicated that miR-145 could ameliorate I/R-mediated Ca^2+^ mishandling via inhibition of CaMKII.

### 3.8. miR-145 Inhibited Inflammatory Response against I/R Injury

**Figure 6:**
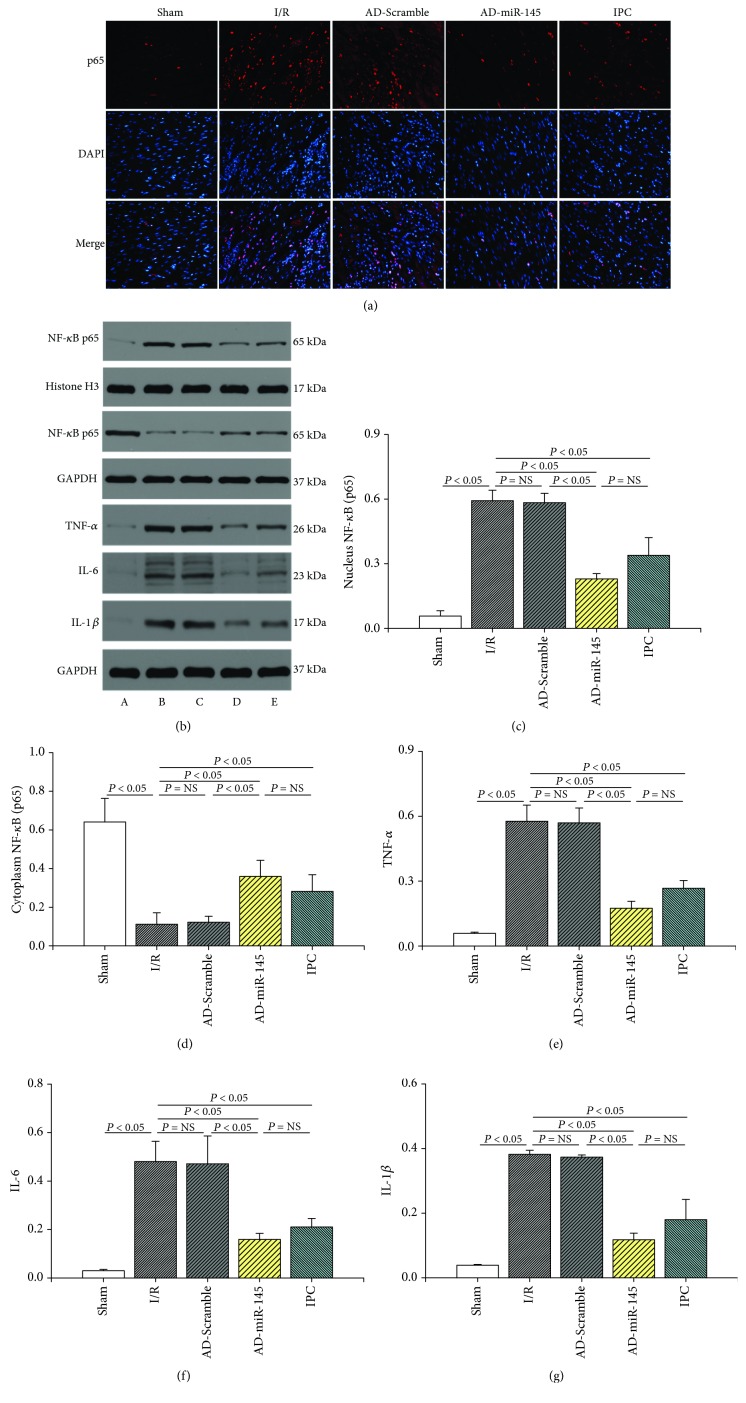
miR-145 inhibited inflammatory response against I/R injury. (a) Representative immunofluorescence images (400x magnification). (b) Representative western blots. (c–g) Quantitative analysis of the immunoreactive band displayed by a bar graph (*n* = 3). (a) Sham group, (b) I/R group, (c) AD-Scramble group, (d) AD-miR-145 group, and (e) IPC group.

Immunofluorescence images and western blotting analyses demonstrated that I/R dramatically increased the nuclear translocation of NF-*κ*B p65 (Figures 6(a)–6(d)) and subsequently led to the release of inflammatory factors, including TNF-*α*, IL-1*β*, and IL-6 (Figures 6(b) and 6(e)–6(g)). Conversely, miR-145 and IPC pretreatment reversed the I/R-induced NF-*κ*B p65 nuclear translocation along with the overexpression of inflammatory factors.

## 4. Discussion

We investigated the cardioprotective mechanism of miR-145 underlying myocardial I/R injury. Our data demonstrated for the first time that overexpression of miR-145 significantly attenuates myocardial I/R injury by reducing cardiomyocyte apoptosis and inhibiting inflammation response. Furthermore, overexpression of miR-145 inhibited the electrophysiological instability and susceptibility to VT in an I/R-injured myocardium. In addition, our results suggested that the protective effects of miR-145 were possibly attributed to inhibition of the CaMKII-mediated ASK1 antiapoptotic signaling pathway and NF-*κ*B p65 anti-inflammatory signaling pathway. Moreover, the cardioprotective activity of miR-145 was proven partially superior to conventional IPC treatment.

Our present study revealed that overexpression of miR-145 alleviates myocardial I/R injury as shown by the diminished release of CK-MB and LDH. The lowered elevation of the ST segment of ECG during the reperfusion period further supported this finding. Moreover, with the prolongation of reperfusion time, the recovery of ST segment improved considerably in the miR-145 and IPC groups. As a kind of free radical scavengers, the activity of SOD is suppressed in response to oxidative stress; on the other hand, the content of MDA, which is a kind of metabolic product of lipid peroxidation [22], will rise. However, overexpression of miR-145 reversed I/R-induced imbalance of SOD and MDA levels, which revealed the potential protective effects of miR-145 against myocardial I/R injury by attenuating oxidative stress.

CaMKII has been shown to exert a detrimental influence on the reversible myocardial IR dysfunction with a prolonged ischemia period [16, 23]. I/R injury and oxidative stress resulted in excessive activation of CaMKII. Subsequently, hyperphosphorylation of RyR2 due to higher activity of CaMKII led to an increase in sarcoplasmic reticulum Ca^2+^ leak and Ca^2+^ oscillations which can trigger spontaneous calcium waves and promote arrhythmogenic Na^+^/Ca^2+^ exchange, finally inducing VT and delayed afterdepolarization [24], whereas inhibition of CaMKII reversed I/R-induced Ca^2+^ oscillations [25]. Our study suggested that an I/R-injured myocardium was more susceptible to VT with a higher expression of ox-CaMKII, p-CaMKII, and p-RyR2 (Ser2814) while upregulation of miR-145 dramatically decreased the activation of CaMKII and RyR2 (Ser2814) thus reducing the risk of arrhythmia. Cordes et al. [12] found that CaMKII was a downstream target gene of miR-145. Moreover, Lee et al. [26] showed that miR-145 suppressed reactive oxygen species-induced Ca^2+^ overload in cardiomyocytes via CaMKII inhibition in vitro, and our research further supported these findings. Therefore, the regulation of CaMKII activity constituted an important mechanism of miR-145-mediated alleviation of the I/R-induced cardiac electrophysiological disturbance.

ASK1 belongs to the mitogen-activated protein kinase family, is highly conserved among different species, and directly regulates cell death through mediation of JNK [27]. Previous studies have demonstrated that ASK1 is prominently activated by I/R stimuli and, subsequently, promotes myocardial apoptosis and necrotic cell death. However, ASK1 knockout mice showed less degree of apoptotic following I/R injury to the heart [28]. CaMKII has been reported to be involved in the development of various cardiovascular diseases and is crucial in the regulation of intracellular calcium cycle [29]. Takeda et al. [30] showed that CaMKII was directly associated with the phosphorylation of ASK1. Moreover, KN93, a CaMKII inhibitor, inhibited Ca^2+^-induced ASK1 phosphorylation, which further confirmed that CaMKII is vital in initiating the phosphorylation of ASK1 at Thr845 [17]. Therefore, inhibiting the overactivation of CaMKII during the I/R process might effectively regulate the Ca^2+^-induced ASK1 signaling pathway and alleviate cardiomyocyte apoptosis. Furthermore, promotion of the opening of the mitochondrial permeability transition pore due to hyperactivation of CaMKII, another leading cause of apoptosis, is also ameliorated [31]. Our study showed that miR-145 was dramatically downregulated by myocardial I/R stimuli, while upregulation of miR-145 effectively inhibited the CaMKII/ASK1 signaling pathway, and these antiapoptotic effects were consistent with the results of Yuan et al. [10]. In addition, the apoptosis index being superior in the miR-145 group than that in the IPC group was also observed in our study, which further verified our hypothesis and confirmed the validity of miR-145 against I/R injury. However, Wu et al. [32] showed that enhanced miR-145 aggravated hypoxia-induced apoptosis in vitro, although the mechanism of miR-145 in the I/R model in vivo was rarely elucidated. We speculated that the varied results may be partly due to different phases at ischemia timing and diverse experimental conditions which may trigger different signaling pathways.

NF-*κ*B was originally identified as a vital transcription factor in both inflammation cells and cardiomyocytes, linking the coordinated inflammatory and cell death signaling pathways [33]. Previous studies have demonstrated that I/R promotes NF-*κ*B nuclear translocation and activates the downstream inflammatory cytokines, such as TNF-*α*, IL-1*β*, and IL-6, while inhibition of NF-*κ*B signal pathways remarkably suppressed the I/R-induced heart damage [34]. In our present work, I/R indeed facilitated the nuclear translocation of NF-*κ*B, and miR-145 overexpression reversed these effects. In addition, the inflammatory cytokines including TNF-*α*, IL-1*β*, and IL-6 in the miR-145 group exhibited lower level expression. These results suggested that the reduction of NF-*κ*B p65 nuclear translocation was induced by the miR-145-mediated CaMKII pathway, which was consistent with ours and others' previous studies [33, 35].

However, there were still some limitations: (1) Although miR-145 presented a stronger inhibitory effect on CaMKII, the risk of malignant arrhythmia and grade of inflammation were not significantly superior to IPC treatment. Compensatory activation of PKA due to CaMKII inhibition might be an alternate explanation [36], as activation of PKA could also result in phosphorylation of RyR2 and intracellular Ca^2+^ mishandling. Further investigations are therefore needed to elucidate the role of PKA during miR-145 treatment; (2) NF-*κ*B could also be activated by ASK1, thus resulting in cardiomyocyte hypertrophy and inflammation response [37]. Therefore, the downregulated ASK1 level may exert a similar inhibition influence on NF-*κ*B-mediated inflammatory cytokine release. Thus, it is necessary to evaluate the interaction between the CaMKII and ASK1 signaling pathways and their modulation on NF-*κ*B nuclear translocation.

## 5. Conclusion

In summary, our present study demonstrated that overexpression of miR-145 had a protective effect against myocardial I/R injury in rat, which may predominantly be due to inhibition of the CaMKII-mediated ASK1 anti-apoptotic pathway and the NF-*κ*B p65 anti-inflammatory pathway. Therefore, miR-145 may be a novel therapeutic target for myocardial I/R injury. Effective regulation of miR-145 alleviates I/R-induced myocardium apoptosis and inflammation as well as the risk of malignant arrhythmia. These cardioprotective effects are expected to improve the prognosis of AMI patients. Moreover, the approach of miR-145 treatment might be partially superior to that of conventional IPC treatment.

## Figures and Tables

**Table 1:** Score criterion of PES.

Score	Score criterion
0	Noninducible preparations
1	Nonsustained tachyarrhythmias induced with three extrastimuli
2	Sustained tachyarrhythmias induced with three extrastimuli
3	Nonsustained tachyarrhythmias induced with two extrastimuli
4	Sustained tachyarrhythmias induced with two extrastimuli
5	Nonsustained tachyarrhythmias induced with one extrastimulus
6	Sustained tachyarrhythmias induced with one extrastimulus
7	Tachyarrhythmias induced during the eight paced beats
8	Cardiac arrest without pacing

**Table 2:** Effects of miR-145 on cardiac function.

	Sham (*n* = 4)	I/R (*n* = 4)	AD-Scramble (*n* = 4)	AD-miR-145 (*n* = 4)	IPC (*n* = 4)
HR (beats/min)	438.25 ± 12.31	430.00 ± 10.29	428.25 ± 11.58	423.25 ± 8.77	428.25 ± 11.06
LVEDd (mm)	5.07 ± 0.15	5.13 ± 0.17	5.15 ± 0.17	5.01 ± 0.18	5.03 ± 0.13
LVEDs (mm)	2.20 ± 0.22	3.30 ± 0.22^∗^	3.27 ± 0.13	3.05 ± 0.13	3.07 ± 0.11
FS (%)	61.37 ± 1.78	34.33 ± 1.29^∗^	35.34 ± 2.09	38.44 ± 1.59^#,&^	40.28 ± 2.92^#^

HR: heart rate; LVEDd: left ventricular end-diastolic dimension; LVEDs: left ventricular end-systolic dimension; FS: fractional shortening. ^∗^*P* < 0.05, compared with the sham group; ^#^*P* < 0.05, compared with the I/R group; ^&^*P* < 0.05, compared with the AD-Scramble group.

## Data Availability

The data used to support the findings of this study are included within the article.
